# Pharmacological Targeting the REV-ERBs in Sleep/Wake Regulation

**DOI:** 10.1371/journal.pone.0162452

**Published:** 2016-09-07

**Authors:** Ariadna Amador, Salvador Huitron-Resendiz, Amanda J. Roberts, Theodore M. Kamenecka, Laura A. Solt, Thomas P. Burris

**Affiliations:** 1 Department of Molecular Therapeutics, The Scripps Research Institute, Jupiter, Florida, 34583, United States of America; 2 Department of Molecular and Cellular Neuroscience, The Scripps Research Institute, La Jolla, California, 92037, United States of America; 3 Department of Pharmacology and Physiology, Saint Louis University School of Medicine, St. Louis, Missouri, 63104, United States of America; McGill University, CANADA

## Abstract

The circadian clock maintains appropriate timing for a wide range of behaviors and physiological processes. Circadian behaviors such as sleep and wakefulness are intrinsically dependent on the precise oscillation of the endogenous molecular machinery that regulates the circadian clock. The identical core clock machinery regulates myriad endocrine and metabolic functions providing a link between sleep and metabolic health. The REV-ERBs (REV-ERBα and REV-ERBβ) are nuclear receptors that are key regulators of the molecular clock and have been successfully targeted using small molecule ligands. Recent studies in mice suggest that REV-ERB-specific synthetic agonists modulate metabolic activity as well as alter sleep architecture, inducing wakefulness during the light period. Therefore, these small molecules represent unique tools to extensively study REV-ERB regulation of sleep and wakefulness. In these studies, our aim was to further investigate the therapeutic potential of targeting the REV-ERBs for regulation of sleep by characterizing efficacy, and optimal dosing time of the REV-ERB agonist SR9009 using electroencephalographic (EEG) recordings. Applying different experimental paradigms in mice, our studies establish that SR9009 does not lose efficacy when administered more than once a day, nor does tolerance develop when administered once a day over a three-day dosing regimen. Moreover, through use of a time response paradigm, we determined that although there is an optimal time for administration of SR9009 in terms of maximal efficacy, there is a 12-hour window in which SR9009 elicited a response. Our studies indicate that the REV-ERBs are potential therapeutic targets for treating sleep problems as those encountered as a consequence of shift work or jet lag.

## Introduction

Periods of sleep and wakefulness are hallmark behaviors of the circadian rhythm. Metabolism is also governed by the circadian rhythm and the secretion of a range of hormones critical for maintaining normal physiological processes are coupled to the circadian clock [1–3]. In fact, circadian disruption is associated with pathology and is linked to myriad diseases including diabetes, obesity, cancer, and sleep and behavioral disorders [4–6]. At the molecular level, the circadian rhythm functions due to the oscillation of core clock proteins. BMAL1 and CLOCK (or NPAS2), components of the positive limb of the clock machinery, heterodimerize and subsequently activate *CRYPTOCHROME* (*CRY*) and *PERIOD* (*PER*) transcription. CRY and PER (negative limb), upon reaching optimal concentration, translocate to the nucleus and inhibit CLOCK/BMAL1 activity, completing the loop [7, 8]. Core circadian clock proteins have been associated with sleep and wakefulness regulation. *Bmal1* deletion in mice results in increased total sleep and sleep fragmentation [9]. Alternatively, mice lacking *Npas2*, exhibit decreased ‘napping’ during the dark period, particularly during the second half, showing a reduction of non-rapid eye movement (NREM) sleep time during the dark phase, compared to wild type mice (WT) [10]. Mice lacking both *Cry1* and *2* present with increased sleep during lights off—about three hours into the dark period [11]. *Per2* mutant mice display increased wakefulness about four hours in advance of the dark phase, consistent with a phase advance observed in motor activity in *Per2* null mice [12]. *Per1/2* deficient mice show no significant difference in 12:12 light:dark entrained conditions, but become arrhythmic in free running settings [13].

Recently, the nuclear receptors REV-ERBα and REV-ERBβ (NR1D1 and NR1D2, respectively) have garnered considerable attention due to their integral roles in regulation of the core clock proteins [14]. REV-ERBs act as transcriptional repressors, inhibiting the transcription of their canonical target genes, including *BMAL1*, *CLOCK*, and *NPAS2* [15–17]. Mice deficient in REV-ERBα present with a shortened period of activity as well as decreased locomotor activity in the second half of the dark period, whereas mice lacking REV-ERBβ, present with a significantly subtler phenotype. However, mice deficient in both REV-ERB proteins display completely disrupted circadian behavior as assessed through locomotor behavior [14]. In a separate study using small molecules targeting REV-ERBs, single injections of REV-ERB agonists, SR9009 or SR9011, at circadian time 6 (CT6) result in loss of locomotor activity in wheel-running cages from CT12 to CT24 under constant dark conditions [18]. Surprisingly, recent studies on REV-ERB regulation of sleep and wakefulness, using the same REV-ERB–specific agonists administered at ZT6, demonstrate immediate increase of REV-ERB-mediated locomotor activity and wakefulness as well as decreased slow-wave sleep (SWS) and rapid-eye movement (REM) sleep in mice both in light:dark and constant darkness conditions for about three hours immediately after injections. Interestingly, the agonists also demonstrated anxiolytic activity in a number of experimental behavioral tests evaluating anxiety [19].

To further investigate the usefulness of REV-ERB agonists to affect sleep characteristics, we administered SR9009 in several experimental paradigms to test short-term tolerance, efficacy, and optimal time points in which SR9009 remained effective. Results from these studies suggest that REV-ERB agonists may be useful therapeutic agents to aid in the maintenance of wakefulness at times when clock resynchronization is necessary, or in order to induce wakefulness in pathological conditions affecting sleep.

## Materials and Methods

### Mice

C57BL6 eight-week old male mice N = 46 were obtained from the Jackson Laboratories (Bar Harbor, ME). All the procedures were conducted in the vivarium at the Scripps Research Institute, which is fully accredited by the Association for Assessment and Accreditation of Laboratory Animal Care (AAALAC), and were approved by the Scripps Institutional Animal Care and Use Committee (IACUC). All experiments were conducted at 22–23°C, humidity of 50–60%. Animals had access to water and regular chow *ad libitum*.

### Compound administration

For all experiments, 8-week-old C57BL6/J male mice were administered SR9009 (100 mg kg^-1^, i.p.) as indicated for EEG assays. The specifics of administration of SR9009 for sleep studies are indicated in the text and figure legends. SR9009 was synthesized as previously described [18] and formulated in 15% cremophor for all animal studies.

### Electroencephalogram (EEG)

EEG analysis was performed as previously described [20–23]. Briefly, stainless steel screw electrodes were implanted under general anesthesia (1–1.5% isoflurane/oxygen vapor mixture) for chronic sleep recording. The electrodes were positioned on the frontal (two electrodes) and parietal (two electrodes) bone over the hippocampus (coordinates: Frontal (0.86mm anterior and 2.0mm lateral to bregma), Parietal: (2.0mm posterior and 2.0mm lateral to the bregma) according to The Mouse Brain in Stereotaxic Coordinates from Franklin and Paxinos, 1997). In order to reduce signal artifacts, one of the electrodes was used as a ground. EEG was recorded in a bipolar arrangement (e.g., frontal vs. parietal cortex). Two wire electrodes were inserted in the neck musculature to record active muscle tone through electromyography (EMG). Insulated leads from the EEG and EMG electrodes were soldered to male pins (220-P02), cemented to the skull with dental acrylic. Mice were allowed 12 days to recover from surgery. For EEG recordings, mice were connected to commutators (PlasticOne) with flexible recording cables allowing them unrestricted movements within the cage and habituated to the recording cages for 72 h. SR9009 was administered as indicated. The EEG and electromyography signals were amplified in a Grass Model 7D polygraph (Quincy, MA), filtered in a frequency range of 0.3 to 60 Hz and sampled at 256 Hz. The EEG and EMG signals were displayed on a computer monitor and stored with a resolution of 128 Hz in the hard drive of a computer for the off-line analysis of the vigilance states, using software supplied by Kissei Comptec (Irvine, CA). The polygraphic results were analyzed semiautomatically by 10-s epochs and classified as wake (W), Slow-Wave sleep (SWS) and Paradoxical Sleep (REM) sleep. The total time of these vigilance states was calculated in minutes within each 1 h period.

### Statistical analysis

**Fig 1:**
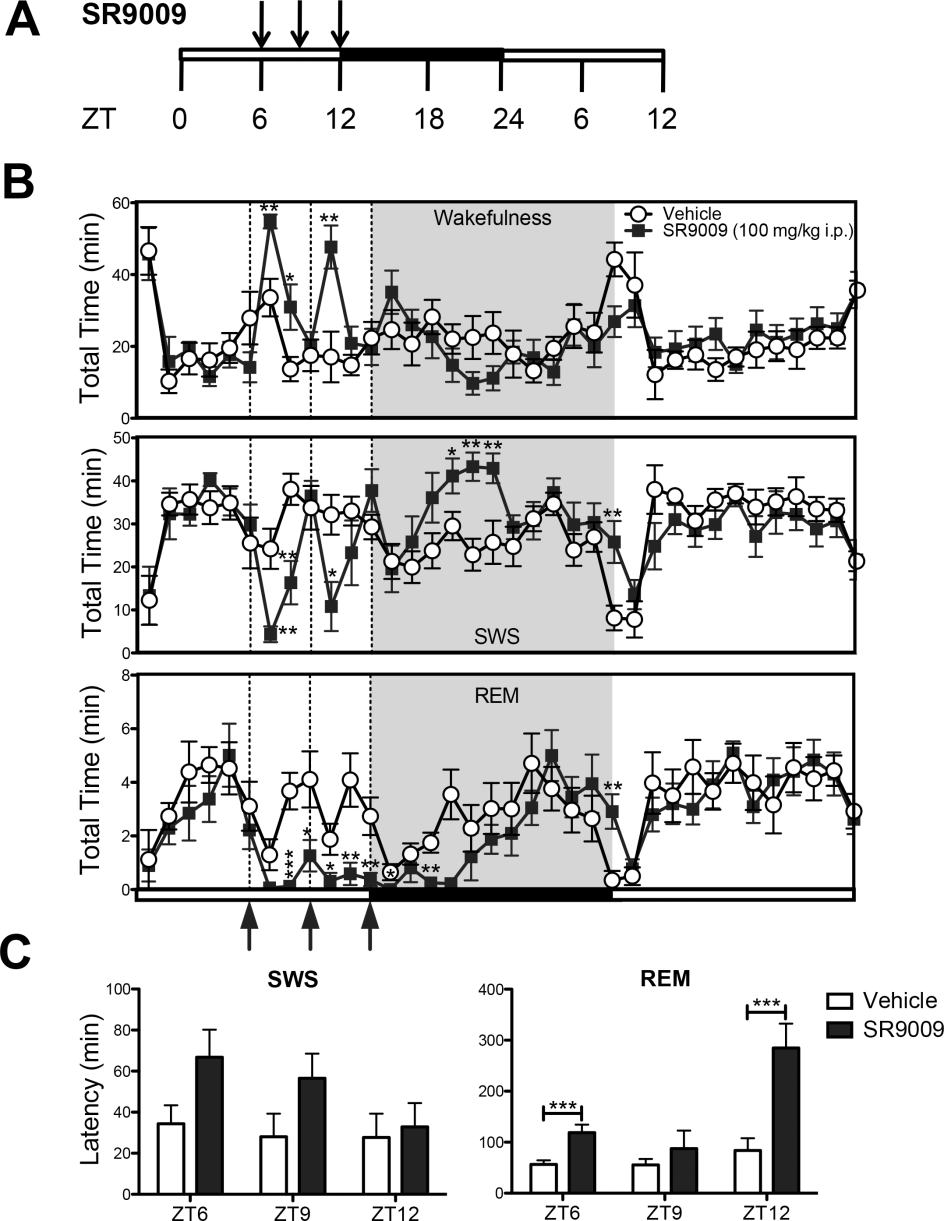
SR9009 remains effective at three-hour interval administration and causes sleep rebound. **(A)** Schematic of experimental paradigm to assess efficacy of the REV-ERB agonist SR9009. Mice were administered with SR9009 at ZT6, ZT9 and ZT12 consecutively in order to assess efficacy of the drug when administered at three-hour intervals. (**B**) SR9009 increases wakefulness when dosed at ZT6 (F(1,14) = 17.3, p<0.005, ZT7 p = 0.002, ZT8 p = 0.03) and ZT9 (F(1,14) = 4.7, p<0.05, ZT10 p = 0.005, ZT11 N.S., but not ZT12 (F(1,14) = 0.54, N.S.). SR9009 inhibits SWS at ZT6 (F(1,14) = 11.0, p<0.01, ZT7 p = 0.001, ZT8 p = 0.004); but not ZT9 (F(1,14) = 1.5, N.S.) and ZT12 (F(1,14) = 0.95, N.S.); and SR9009 inhibits REM sleep at ZT6 (F(1,14) = 16.0, p<0.005, ZT8 p = 0.0002, ZT9 p = 0.001), ZT9 (F(1,14) = 13.9, p<0.005, ZT10 p = 0.03, ZT11 p = 0.006, ZT12 p = 0.007), and ZT12 (F(1,14) = 7.7, p < 0.05, ZT13 p = 0.04, ZT15 P = 0.007). There was no rebound in wakefulness (F(1,14) = 2.7, N.S.) or REM (F(1,14) = 8.9, N.S.) during the following dark phase, but there was a significant SWS rebound (F(1,14) = 13.7, p<0.005, ZT16 p = 0.004, ZT17 p = 0.001, ZT18 p = 0.01, and ZT24 p = 0.007). (**C**) SR9009 significantly increased REM sleep latency (F(1,8) = 6.8, p<0.05) at ZT6 (p = 0.003) and ZT12 (p = 0.002). *P<0.05, ** *P*<0.01, *** *P*<0.005.

For the first study in which SR9009 was administered in the same mice (N = 8 per group) at ZT6, 9, and 12, two-way ANOVAs were performed using time spent in each vigilance state over the 3 h following each injection to determine the acute effects of the drug. In order to evaluate drug rebound effects, ANOVAs were performed using the next 12 h phase following injections. For the second study in which SR9009 was administered at ZT6 for 3 consecutive days and for the third study in which SR9009 effects were examined at 11 different time points across the circadian cycle, smaller groups sizes were used (N = 4). In these cases we present the time course data, but use non-parametric Mann-Whitney *U* tests in order to best examine the drug effects in these smaller group size experiments. Based on the data presented in Fig 1 and previous experience with SR9009, time spent in each vigilance state in the two hours following injections were summed for these analyses. The p-value of <0.05 was set for rejection of the null hypothesis. For latency assessment (time to SWS or REM), values were analyzed using ANOVA and posthoc Bonferroni tests for significance. All data are expressed as the mean ± s.e.m. The results of the statistical tests are detailed in the figure legends. Mice were randomly assigned to each group.

## Results

### Repeated acute administration of SR9009 effectively induces wakefulness in mice during the light period

We previously demonstrated that acute administration of SR9009 at ZT6 (peak *REV-ERBα* mRNA expression) results in increased wakefulness and decreased SWS and REM sleep immediately after injections [19]. The duration of induction of wakefulness was approximately two to three hours and it was unclear if this was due to the pharmacokinetics of the drug, desensitization of the effect, or decrease in the expression of the target due to its circadian pattern of expression. We hypothesized that a repeated dosing schedule would aid in our assessment of the relatively short duration of action of SR9009. We injected mice with SR9009 (100mg/kg) or vehicle at ZT6, ZT9, and ZT12 and recorded their sleep sleep/wake patterns via EEG (Fig 1A). Dosage and timing was based on previously published data indicating that this dose exhibited adequate brain exposure, resulted in maximal repression of target genes *in vivo*, and acute effects last approximately two to three hours [18, 19]. Mice showed increased alertness in response to the drug at ZT6 and ZT9, but not ZT12, decreased SWS only following the ZT6 injection, but decreased REM following all three drug administrations (Fig 1B). Additionally, the sleep disruptive behavior of the drug did not extend into the next light period, as the mice sleep pattern appears to synchronize to their regular wake/sleep pattern. Repeated SR9009 administration effectively increased the time required for the mice to recover their REM sleep behavior, which we have labeled ‘latency’ (Fig 1C), as sleep remains suppressed for an extended period of time after injections (Fig 1B). These results suggest that therapeutic targeting of the REV-ERBs may assist in maintaining wakefulness and repressing sleep in a sustained manner when administered throughout the light period.

### REM-inhibiting effects of SR9009 are maintained upon repeated daily dosing in mice

**Fig 2:**
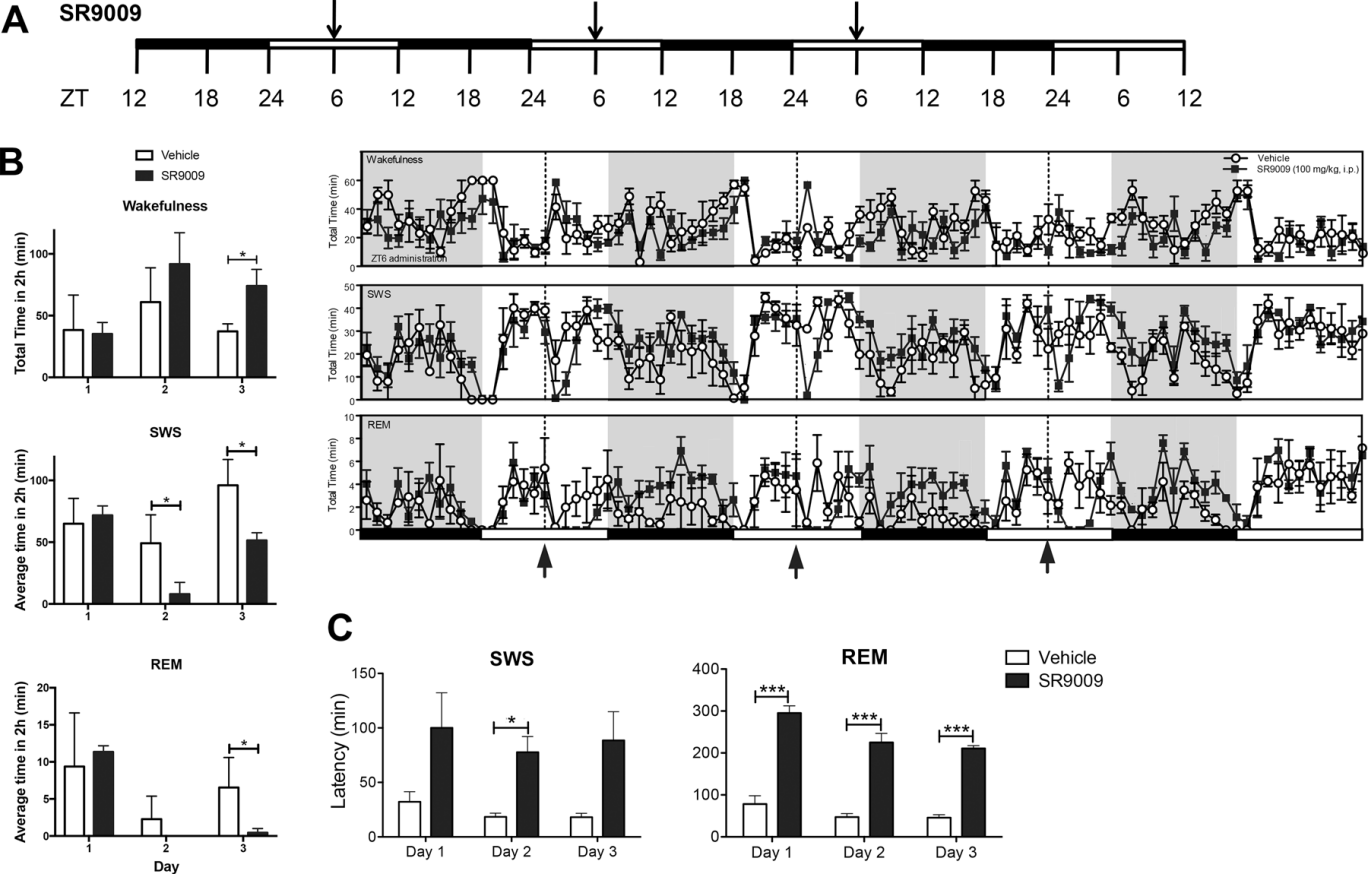
**Acute daily administration of SR9009 does not induce short-term tolerance** (**A**) Schematic of experimental paradigm to assess short-term tolerance in response to the REV-ERB agonist SR9009. Mice were injected with SR9009 (i.p. 100mg kg^-1^) at ZT6 on three consecutive days in order to assess tolerance. (**B**) SR9009 increased wakefulness, but only reached significance on day 3 (p<0.05), decreased SWS on days 2 and 3 (p<0.05), and decreased REM on day 3 (**C**) Overall, SR9009 increased SWS latency (F(1,5) = 7.7, p<0.05, only significant on Day 2 p = 0.02). SR9009 increased REM sleep latency, reaching significance on all days tested (F(1,5) = 183.4, p<0.0001, Day 1 p = 0.0004, Day 2 p<0.0001, and Day 3 p<0.0001). *P<0.05, *** *P*<0.005.

Decreased responsiveness to drugs, also known as tolerance or tachyphalaxis, may occur with repeated administration and is commonly associated with drugs that alter sleep and wakefulness. Tachyphalaxis can limit the utility of various classes of drugs. We sought to determine if tolerance to SR9009 could occur in a short-term paradigm where mice were administered SR9009 once per day for 3 days (Fig 2A). Mice were administered SR9009 [100mg/kg] or vehicle at ZT6, the optimal efficacy dosing time for wake induction, for three consecutive days. Mice showed increased wakefulness (day 3), decreased SWS (days 2 and 3) and REM (days 3) sleep following drug administration (Fig 2B). Latency to re-enter REM sleep was significantly increased on each of the days tested, (Fig 2C) and SWS was significantly increased on day 2. These results indicate that regular administration of SR9009 may be useful to reduce sleep with a low probability of developing short-term tolerance. However, the wake-inducing effect of the drug was less evident on each day tested although the effect on suppressing SWS and REM sleep appears to be maintained.

### SR9009 decreases REM sleep throughout the light period

**Fig 3:**
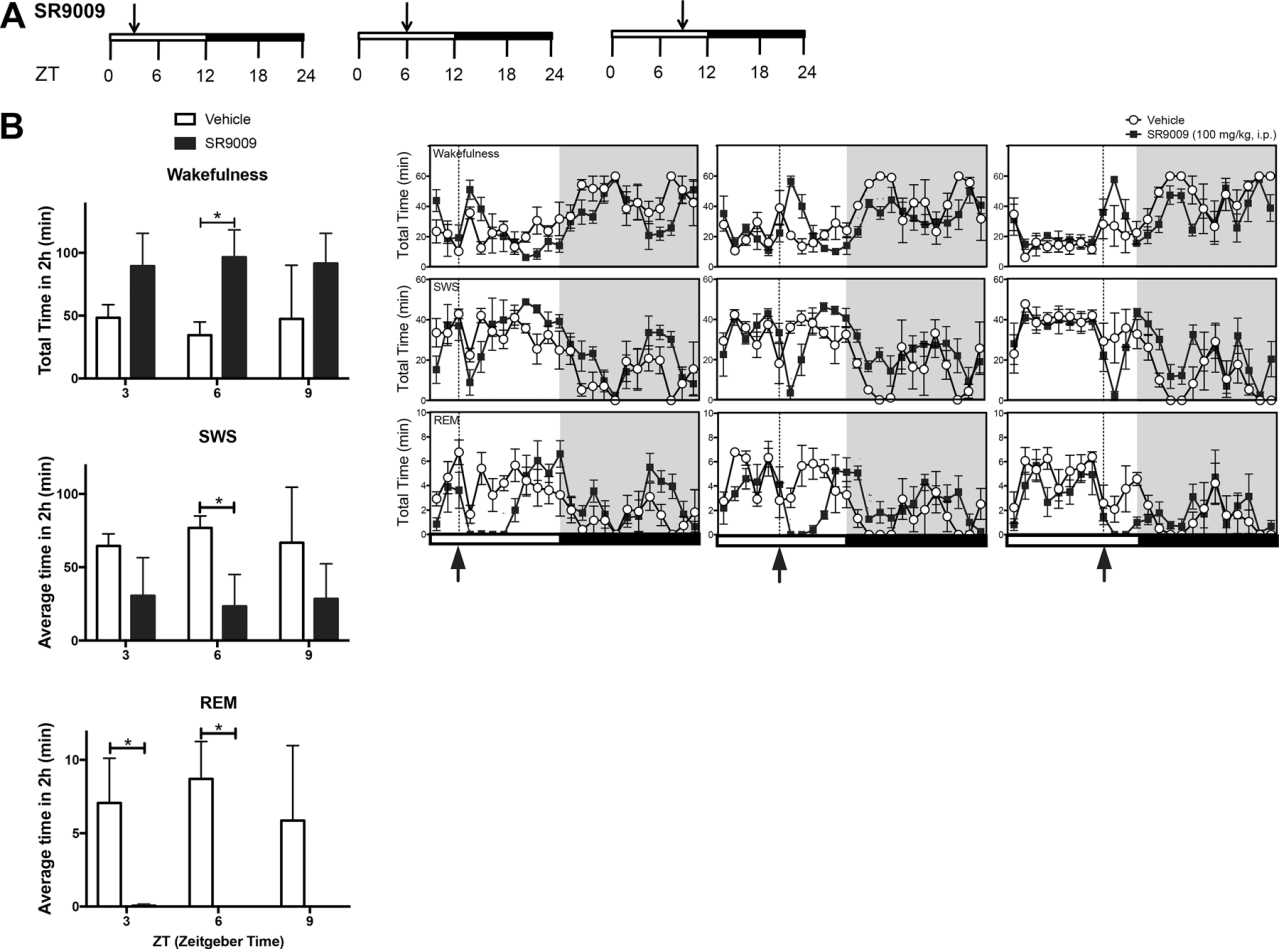
Time-Response EEG activity of SR9009 during the light phase. (**A**) Schematic of experimental paradigm to assess SR9009 time-dependent behavioral activity of mice EEG. Mice were dosed with SR9009 at different time points (ZT3, ZT6, and ZT9) on separate days in order to generate a Time-Response Curve. (**B**) Injections with SR9009 (i.p. 100mg kg^-1^) induced wakefulness at ZT6 (p<0.05), but not ZT3 and ZT9, reduced SWS at ZT6 (p<0.05), but not ZT3 and ZT9, and decreased significantly REM sleep at ZT3 (p<0.05), and ZT6 (p<0.05), but not at ZT9. The maximal effect on wakefulness was achieved when the animals were dosed at ZT6 compared to ZT3 and ZT9. *P<0.05

Chronopharmacology is becoming a more prominent subject as maximization of drug efficacy may be related to timing of drug administration in relation to the target’s circadian rhythm of expression [24, 25]. Given the circadian pattern of expression of the REV-ERBs, we sought to investigate the time-dependent effects of SR9009 administration on sleep and wakefulness. To cover a 24-hour period, we administered SR9009 at three-hour time points starting at ZT0 (lights on). Two cohorts of mice were used for this, with cohort 1 used for ZT0, ZT6, ZT12, and ZT18 and cohort 2 used for ZT3, ZT9, ZT15, and ZT21. Injections were done 7–10 days apart. SR9009 induced wakefulness and inhibited SWS at ZT6 and inhibited REM sleep at ZT3 and ZT6 (Fig 3A and 3B). Drug efficacy increased up to ZT6 and decreased after this time point, with maximal effect occurring at ZT6, which is consistent with peak *REV-ERB* mRNA expression. Interestingly, while SR9009 treated mice return to their sleep routine during their nocturnal period, a rebound effect appeared to occur where mice had decreased wakefulness and increased SWS and REM sleep following the second and third injections (Fig 3B). These data indicate that the REV-ERB agonist SR9009, administered at different times of the light period, can be effective for inducing wakefulness and decreasing sleep, making it a useful therapeutic tool for wake stimulation.

### SR9009 does not augment wakefulness throughout the dark period

**Fig 4:**
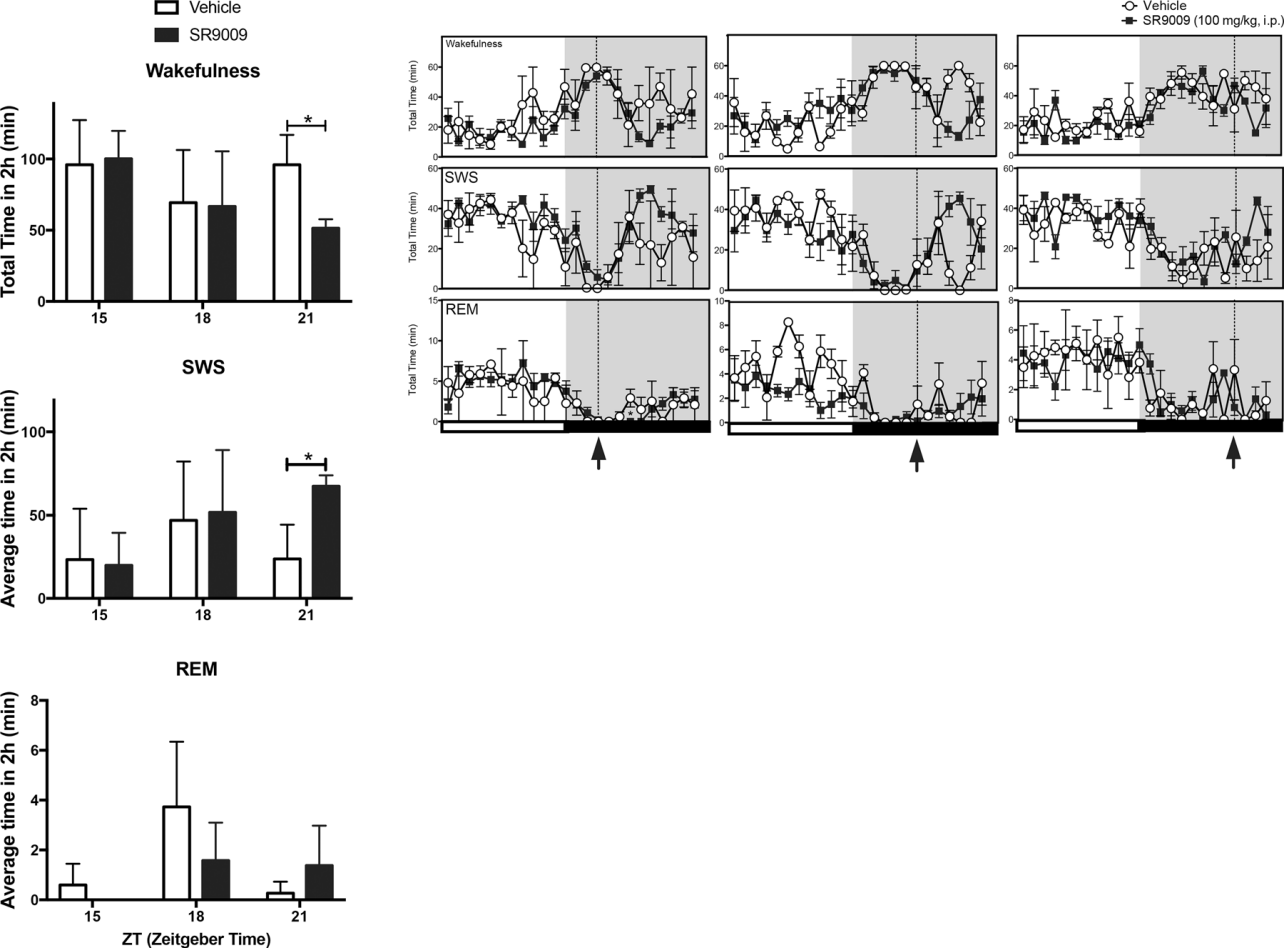
Time-Response EEG activity of SR9009 during the dark phase Mice were dosed with SR9009 at different time points (ZT15, ZT18, ZT21) on separate days in order to generate a Time-Response Curve. Injections with SR9009 (i.p. 100mg kg^-1^) failed to induce wakefulness at ZT15 and ZT18. but rather decreased wakefulness at ZT21 (p<0.05). SR9009 increased SWS at ZT21 (p<0.05) and had no effect on REM at any of these time points. *P<0.05.

Since REV-ERB expression diminishes during the wake period in mice, we wanted to assess the effect of SR9009 during the dark phase in mice. W injected SR9009 at different three-hour time points starting at lights off (ZT12). SR9009 failed to increase wakefulness at all time points tested during the lights off phase, which was not unexpected, given the mice were already awake and likely have reached a near maximal level of wakefulness during this period. Surprisingly, while not significant using the first 2 h following drug administration at ZT18 in the analysis, it appeared that SR9009 inhibited wakefulness rather than increasing it 4–6 hr post administration (Fig 4A). At ZT21, the 2h post-drug administration analysis showed significant wake decrease and SWS increase due to SR9009 administration (Fig 4). REM sleep was also increased at ZT21 even though it failed to reach significant levels. Sleep-need accumulation seems unlikely in this scenario, since the SR9009-injected animals did not appear ‘more awake’ than their vehicle counterparts.

### SR9009 affects light:dark and dark:light transitions

**Fig 5:**
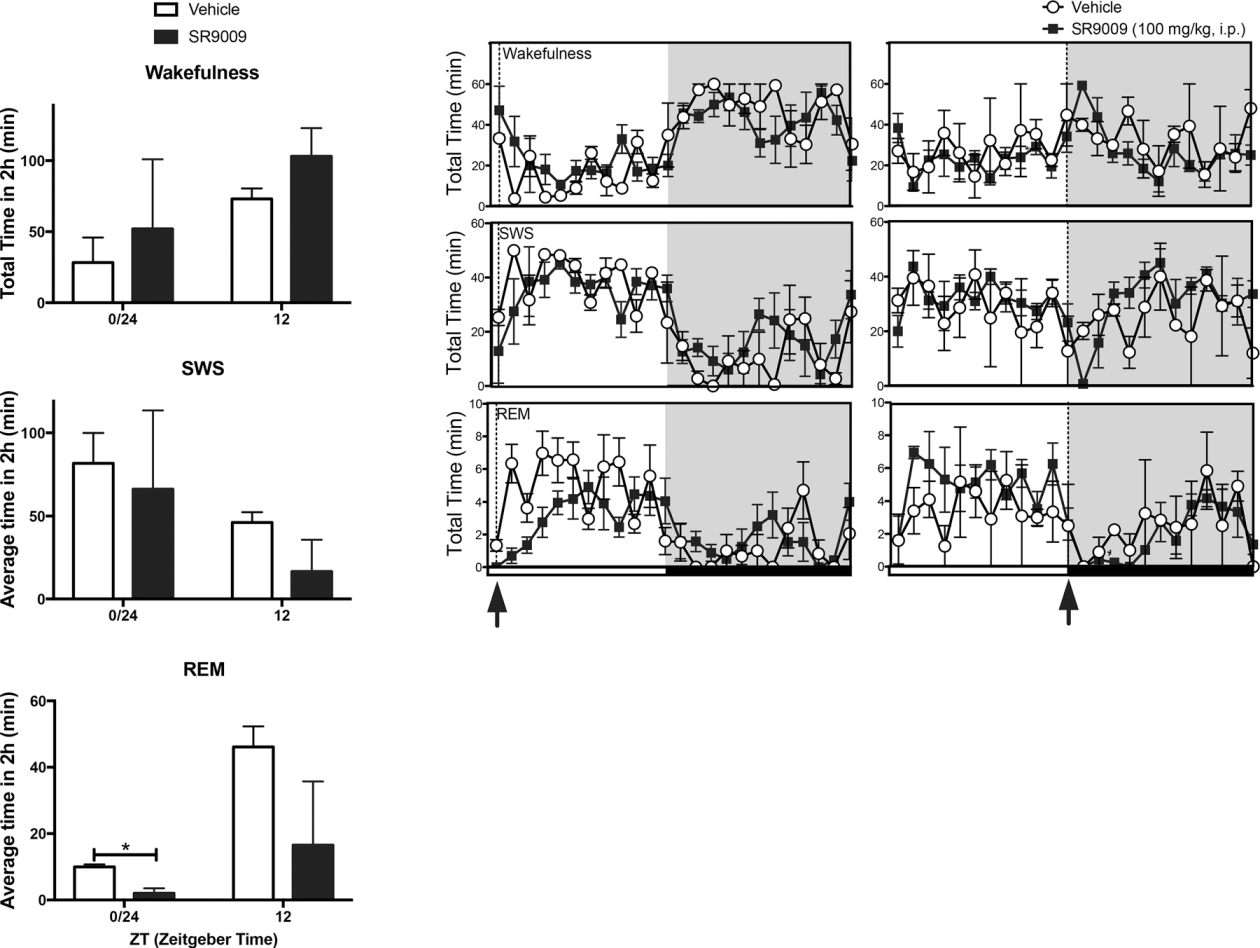
SR9009 effect during transitions from light-to-dark and dark-to-light. There was no effect of SR9009 on wakefulness or SWS at ZT0/24; however REM was significantly decreased at this time (p<0.05). There were trends toward increased wakefulness and decreased SWS and REM by SR9009 at ZT12 (all p’s = 0.06), but did not reach statistical significance. *P<0.05.

Light is the main zeitgeber that synchronizes the master clock located in the suprachiasmatic nucleus (SCN) and adjusts the endogenous clock to an exactly 24-hour cycle [26]. Transitions of dark to light and dark to light are pace-making events that either properly synchronize the endogenous molecular machinery with the environment, or critically alter the internal clock when signaling is incoherent with the ongoing internal molecular machinery, for example, when traveling across time zones. We wanted to assess whether SR9009 could act as a zeitgeber disruptive/enhancing agent at light dark transition periods. SR9009 was administered at ZT0 and ZT12, lights on and lights off, respectively. When administered at ZT0, the time point in which the mice would be signaled to sleep, SR9009 significantly reduced REM sleep, suggestsuggesting that REV-ERB targeting with a more potent drug may be used as a non-photic zeitgeber to extend the activity period. When SR9009 was administered at the transition from light to dark (ZT12), it also increased wakefulness, reduced SWS and REM sleep compared to vehicle controls (Fig 5) but failed to reach singificance. Regardless of the strong environmental cues of dark and light, SR9009 shows a tendency to reduce REM sleep at light:dark transitional time points.

### Latency to re-enter REM sleep after SR9009 administration shows a circadian pattern

**Fig 6:**
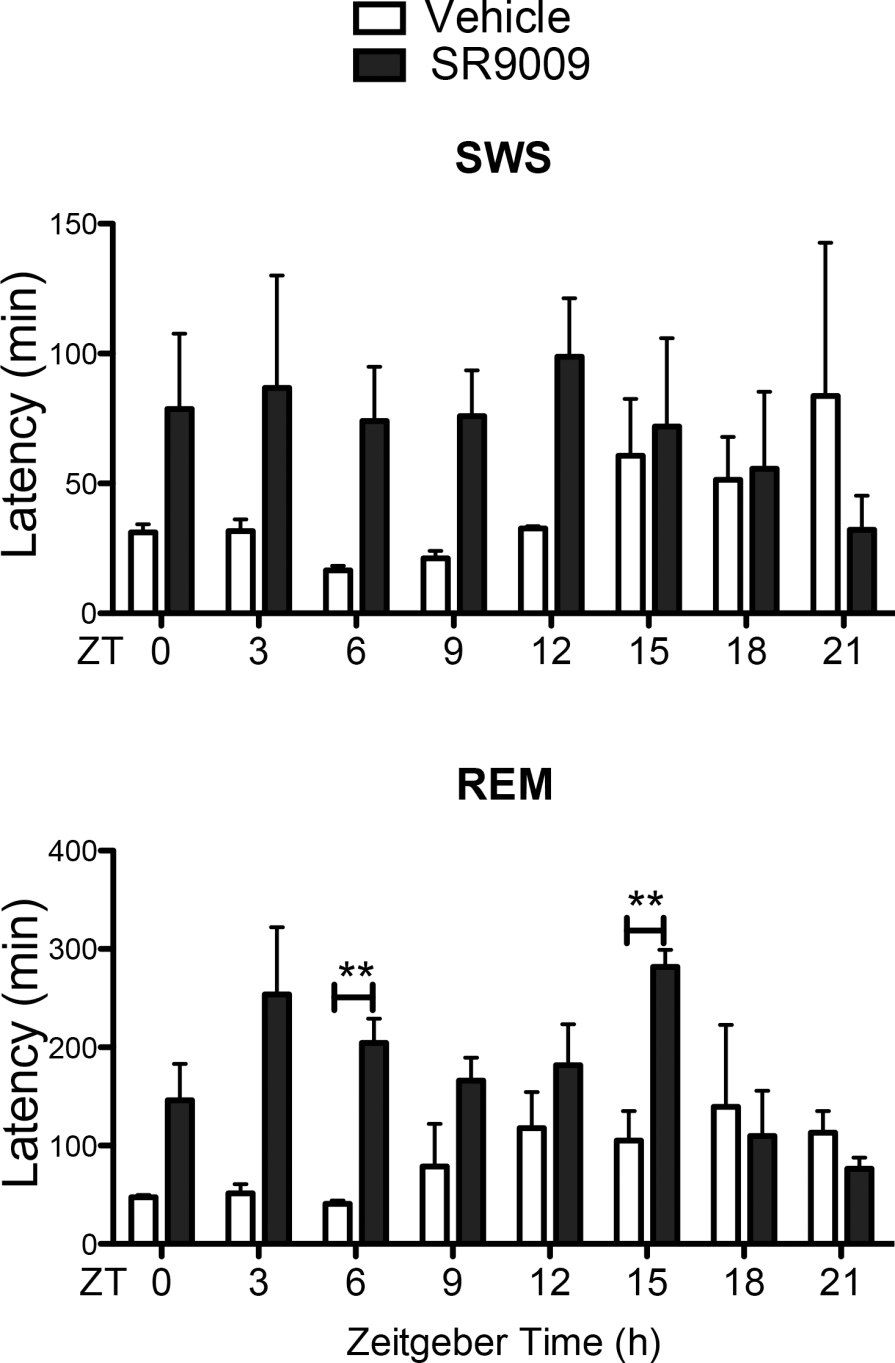
SR9009 SWS and REM sleep latency during a 24h period. SR9009 injections increased the latency to enter REM sleep (F(1,4) = 16.9, p<0.05, ZT6 p = 0.003, ZT15, p = 0.005) in a circadian manner. The effects on SWS were not significant in the overall ANOVA. Latencies to enter SWS and REM following the acute injections at the three-hour intervals presented in Figs 3–6 are graphed across a 24h period. ** *P*<0.01.

Chronopharmacological targeting of REV-ERBs using agonist SR9009 increased wakefulness during the lights on period and increased latency to reach pre-administration levels of REM sleep. The latency data was summarized in Fig 6, which highlights that both SWS and REM sleep are affected by SR9009 selectively along the light period, once more suggesting that the drug effect is REV-ERB mediated. The lights-off period shows significant REM latency increase at ZT15 only. Time point administration thereafter showed no latency effects of SR9009 on SWS or REM sleep.

## Discussion

Our studies aimed at expanding our understanding of the utility of REV-ERB agonists as pharmacological agents. In the present report, we characterized the effects of pharmacologically activating REV-ERB on sleep and demonstrated that SR9009 induces wakefulness at different times during the light period and shows no short-term tolerance development while maintaining its efficacy when administered repeatedly throughout the rodent’s inactive period. Our results suggest that pharmacological targeting of REV-ERB may aid in maintaining wakefulness when the drive for sleep is significant including shift work and jet lag. A recent study on the role of REV-ERBα in sleep regulation showed that this REV-ERB is involved in wake repression in the last hours of the murine inactive period (ZT10-ZT12) and participates in sleep homeostasis [27]. In this report we targeted both REV-ERBs with dual agonist SR9009 and showed that at ZT9 targeting results in increased wakefulness. While these phenotypes appear at odds with each other, further characterization of the drug interaction with REV-ERBα individually *in vivo* may be necessary. Drug treatment and Time Curve assessment of the drug in individual and double KO mouse models may be a next step to unveil the mechanistic pathway of the drug.

The number of compounds that have been developed to target components of the circadian molecular clock is very limited and of those there is even less data available demonstrating a clear therapeutic value for these types of compounds at this point [28]. The REV-ERBs have been proposed as core clock proteins since mice lacking both REV-ERBα and REV-ERBβ lack a circadian rhythm [14]. Since the REV-ERBs are members of the nuclear receptor superfamily, they are ligand-regulated transcription factors and thus are viable targets for evaluation of their pharmacological potential. Small molecule ligands specific to the REV-ERBs have been identified and characterized and were recently used to assess the role of REV-ERB on wake/sleep and anxiety behaviors [18, 19]. SR9009 and SR9011, administered at ZT6, the time of peak REV-ERB mRNA expression, increased wakefulness for the first two to three hours immediately after injection in EEG studies.

Our work assessed the efficacy of the drug when administered at multiple times during the day in order to determine optimal and suboptimal dosing times. Interestingly, the drug remained effective when administered intermittently during the light phase. The magnitude and duration of these effects gradually decreased with increasing distance from the time point of ZT6, which is consistent with the circadian property of the target protein. Surprisingly, at ZT18 and ZT21, SR9009-mediated decreased wakefulness differs from effects previously observed in our laboratory when SR9011, a less potent yet similar REV-ERBα agonist, was used at ZT18 (See Banerjee *et al*. *Nat Commun*, 2014) [18, 19]. These results were further replicated with another cohort. SR9009 differs only slightly from SR9011 as SR9009 has a urea instead of a carbamate group [18]. However, such structural differences may cause differential interactions with the receptors, which may help explain varying effects on sleep and wakefulness. Alternatively, the results observed with SR9009 may be a consequence of off-target effects. However, these compounds were evaluated in a host of assays to determine this and to date, we have found little–to—no cross reactivity with these compounds at other nuclear receptors, G-protein coupled receptors, or ion channels tested [18, 19]. While further characterization of the drugs is warranted, the effects are likely a consequence of the different pharmacokinetic/pharmacodynamics properties between the two compounds [18]. Regardless, the magnitude and efficacy of REV-ERB agonists seem to be time-dependent, as expected from a drug targeting a circadian molecule (Fig 6). In fact, the optimal time for inducing wakefulness was ZT6, consistent with the drug acting via REV-ERB, a circadian molecule that maximizes its transcript at ZT6 and its protein at ZT8 [29]. An increasing number of drugs have been demonstrated to vary their effects according to the time of administration [30, 31]. In fact, drug metabolism increases during the wake period, as most of the drug-metabolizing enzymes are at peak levels [32]. Factors such as drug absorption, drug distribution by blood flow, drug metabolism by liver enzyme availability or activity, among others, may determine time-dependent efficacy [30]. Additionally, sleep-inducing drugs like benzodiazepines and Z-drugs show chronopharmacological effects, with benzodiazepines appearing to disturb the circadian clock [33–37]. Moreover, toxicity and side effects seem to be highly time dependent, indicating that timing of drug administration is a key point to consider to reduce or take advantage of such effects [38].

SR9009 shows rebound effects, which will need further characterization. While sleep rebound in a sleep drug may seem limiting, SR9009 rebound is limited to the next twelve-hour period. A rebound during the activity period for a drug that increased wakefulness during the previous sleep phase may signal homeostatic response, a healthy response to sleep disruption. Importantly, the drug does not affect the next sleep/wake cycle, thus, not dragging its effect further than the next 12h period. The applicable scenarios (e.g. jetlag, shiftwork) are yet to be tested. But in such cases, sleep homeostasis seems like a favorable and desired scenario.

Disruption in circadian behaviors, particularly sleep, is associated with negative health outcomes. Insufficient sleep contributes to obesity [39], diabetes [40], cardiovascular disease [41], and decreased alertness and memory [42]. In a recent study, patients exposed to one week of insufficient sleep (5.7 ±0.03 h) revealed up and down regulated genes, including those involved in the circadian rhythm, sleep homeostasis, oxidative stress and metabolism [43], which highlights the crosstalk between sleep and metabolism.

Tachyphylaxis has been observed in wake-inducing drugs, such as amphetamines and modafinil [44, 45]. Importantly, targeting the REV-ERBs poses a unique additional challenge. Within the promoter of the *REV-ERBα* gene is a REV-ERB response element and, since the REV-ERBs are transcriptional repressors, activation of the receptor by agonists may reduce its own expression. Thus, we thought it important to examine the efficacy of repeated daily dosing of SR9009 to determine if there is loss of effectiveness. This was not observed indicating that tachyphylaxis is not an issue at least in terms of repeated dosing over 3 days. Importantly, when the drug was administered continuously at three-hour intervals, the effect of the drug at ZT9 diminished compared to ZT6, and ZT12 administration showed even less response compared to ZT9. This may be indicative of tolerance issues. However, as the drug targets a circadian molecule, which is reducing its availability from ZT6 to ZT12, such decreased response to the drug may be due to lack of target rather than tolerance development.

Methamphetamines and cocaine, drugs of abuse, lead to tolerance development, by which the organism needs higher doses of the same stimulus to obtain similar effects to the initial dose [46, 47]. Recent studies have demonstrated that REV-ERB agonists not only induced wakefulness and reduced sleep, the molecules were also effective as anxiolytic agents [19]. FDA-approved drugs for reducing anxiety normally reduce wakefulness, e.g. benzodiazepines [48, 49]. Moreover, few drugs in the market induce wakefulness and reduce sleep, e.g. amphetamines, armodafinil, and modafinil [50–52], and none have yet been approved to treat jet lag. Moreover, safety is a concern with the use of these drugs. Amphetamines, cocaine and even caffeine cause addiction and are considered drugs of abuse [53–57]. REV-ERB agonists are unique therapeutic tools to target sleep, potentially reducing or even eliminating addictive and anxiogenic side effects [19]. The recent literature shows that the REV-ERB agonist SR9011 appeared to reduce the rewarding properties of cocaine, an effect similarly observed with modafinil [58, 59]. However, unlike modafinil [44], SR9011 failed to show aversive behavior in mice. Interestingly, REV-ERBα represses the transcription of the tyrosine hydroxylase gene [60] while the mechanism of activity of modafinil, cocaine and amphetamines involves blocking the dopamine transporter (DAT), thus inhibiting the uptake of dopamine from the synaptic cleft and increasing dopaminergic activity. Interestingly, modafinil selectively targets DAT while the other psychostimulants show promiscuity with all monoamine transporters [61–66], which may partially explain conflicting results in modafinil’s potential for addiction [59, 67, 68]. More extensive studies are necessary to establish REV-ERB’s role and interaction with the monoaminergic system. Since addiction and tolerance are closely related, short-term potential tolerance problems were also assessed and our results showed that the drug is still efficient at reducing SWS and REM sleep when dosed in a daily manner for three consecutive days using a consistent drug concentration. Additional studies need be performed to assess long-term drug efficacy.

SR9009 appears to mediate two separate behaviors depending on the light status. During the light phase and during transitional time points (ZT12 and ZT24), the drug induces wakefulness immediately post administration. However, decreased wakefulness observed in the dark phase does not occur immediately after injections compared to lights-on period in which wakefulness induction occurs right after injections. Rather there is latency that precedes this effect, which is shorter as the injection approaches ZT24.

In conclusion, our work demonstrates that REV-ERBs may be used as a novel target to modulate sleep/wake aberrant behavior. Further characterization of REV-ERB agonists is necessary to assess how effective these compounds may be in more challenging scenarios that mimic those of exposure to traveling across time zones and those of shift work. However, our results establish the groundwork for these studies involving the treatment of shift work, jet lag, sleep disorders, as well as other neuropathies that affect sleep and wakefulness. Pathological sleep problems such as those observed in narcolepsy and other diseases may also be a target for this class of compounds.
